# Analysis of SDHAF3 in familial and sporadic pheochromocytoma and paraganglioma

**DOI:** 10.1186/s12885-017-3486-z

**Published:** 2017-07-24

**Authors:** Trisha Dwight, Un Na, Edward Kim, Ying Zhu, Anne Louise Richardson, Bruce G. Robinson, Katherine M. Tucker, Anthony J. Gill, Diana E. Benn, Roderick J. Clifton-Bligh, Dennis R. Winge

**Affiliations:** 1 Cancer Genetics, Hormones and Cancer Group, Kolling Institute of Medical Research, Royal North Shore Hospital, Sydney, 2065 Australia; 2 University of Sydney, Sydney, 2006 Australia; 3 Department of Medicine, University of Utah Health Sciences Center, Salt Lake City, UT 84132 USA; 4 Department of Biochemistry, University of Utah Health Sciences Center, Salt Lake City, UT 84132 USA; 5 Hunter New England Health, Royal North Shore Hospital, Sydney, 2065 Australia; 6 Hereditary Cancer Service, Prince of Wales Hospital, Sydney, 2031 Australia; 7 Department of Anatomical Pathology, Royal North Shore Hospital, Sydney, 2065 Australia; 8 Northern Cancer Translational Research Unit, Royal North Shore Hospital, Sydney, 2065 Australia

**Keywords:** Succinate dehydrogenase, SDHAF3, SDHB, Pheochromocytoma, Paraganglioma

## Abstract

**Background:**

Germline mutations in genes encoding subunits of succinate dehydrogenase (SDH) are associated with the development of pheochromocytoma (PC) and/or paraganglioma (PGL). As assembly factors have been identified as playing a role in maturation of individual SDH subunits and assembly of the functioning SDH complex, we hypothesized that SDHAF3 variants may be associated with PC/PGL and functionality of SDH.

**Methods:**

DNA was extracted from the blood of 37 individuals (from 23 families) with germline SDH mutations and 18 PC/PGL (15 sporadic, 3 familial) and screened for mutations using a custom gene panel, containing SDHAF3 (SDH assembly factor 3) as well as eight known PC/PGL susceptibility genes. Molecular and functional consequences of an identified sequence variant of SDHAF3 were assessed in yeast and mammalian cells (HEK293).

**Results:**

Using massively parallel sequencing, we identified a variant in SDHAF3, c.157 T > C (p.Phe53Leu), associated with increased prevalence in familial and sporadic PC/PGL (6.6%) when compared to normal populations (1.2% [1000 Genomes], *p* = 0.003; 2.1% [Exome Aggregation Consortium], *p* = 0.0063). In silico prediction tools suggest this variant is probably damaging to protein function, hence we assessed molecular and functional consequences of the resulting amino acid change (p.Phe53Leu) in yeast and human cells. We showed that introduction of SDHAF3 p.Phe53Leu into Sdh7 (ortholog of SDHAF3 in humans) null yeast resulted in impaired function, as observed by its failure to restore SDH activity when expressed in Sdh7 null yeast relative to WT SDHAF3. As SDHAF3 is involved in maturation of SDHB, we tested the functional impact of SDHAF3 c.157 T > C and various clinically relevant SDHB mutations on this interaction. Our in vitro studies in human cells show that SDHAF3 interacts with SDHB (residues 46 and 242), with impaired interaction observed in the presence of the SDHAF3 c.157 T > C variant.

**Conclusions:**

Our studies reveal novel insights into the biogenesis of SDH, uncovering a vital interaction between SDHAF3 and SDHB. We have shown that SDHAF3 interacts directly with SDHB (residue 242 being key to this interaction), and that a variant in SDHAF3 (c.157 T > C [p.Phe53Leu]) may be more prevalent in individuals with PC/PGL, and is hypomorphic via impaired interaction with SDHB.

**Electronic supplementary material:**

The online version of this article (doi:10.1186/s12885-017-3486-z) contains supplementary material, which is available to authorized users.

## Background

Succinate dehydrogenase (SDH) plays an integral role in the tricarboxylic acid (TCA) cycle, where it couples the oxidation of succinate to fumarate to ubiquinone reduction. SDH is comprised of four nuclear encoded subunits (SDHA, B, C and D), and germline mutation in any of these SDH subunits is associated with a variable risk of developing neoplasia [1]. Nevertheless, discordant phenotypes are observed both within and between families carrying the same *SDH* mutation, suggesting that other environmental, genetic or epigenetic factors influence the clinical phenotype.

SDH genes (*SDHA*, *SDHB*, *SDHC*, *SDHD*) act as classical tumor suppressors, such that germline heterozygous inactivating mutations coupled with somatic loss of the remaining wild-type allele leads to complete loss of enzyme function and development of associated tumors. SDHx mutations have been linked to tumorigenesis as a result of a number of downstream consequences. SDH deficiency results in succinate accumulation, due to inability of SDH to catalyze the oxidation of succinate to fumarate. In turn, the elevated succinate can inhibit α-ketoglutarate-dependent dioxygenases, resulting in pseudo-hypoxia and hypermethylation of histones and DNA. Inhibition of the α-ketoglutarate-dependent dioxygenase, prolyl hydroxylase (PHD), leads to HIF stabilization, increased expression of HIF targets, and ultimately induction of a hypoxic response under normoxic conditions (pseudo-hypoxia). In line with this proposed mechanism of action, both increased stability of HIF and increased expression of HIF targets have been identified in *SDHx*-mutated paragangliomas and pheochromocytomas [2–4]. Additionally, accumulated succinate inhibits other α-ketoglutarate-dependent dioxygenases, such as histone demethylases of the Jumonji demethylase family and TET hydroxylases, resulting in hypermethylation of histones and DNA. This mechanism has been observed in *SDHx*-mutated paragangliomas, pheochromocytomas, and gastrointestinal stromal tumors [5–8].

Recently, SDH assembly factors (SDHAF1, SDHAF2, SDHAF3 and SDHAF4) have been identified as being crucial for maturation and effective functioning of SDH within mitochondria [9–11]. To date, mutations affecting *SDHAF1* (involved in maturation of SDHB) and *SDHAF2* (required for covalent attachment of FAD to SDHA) have been associated with human diseases. *SDHAF1* mutations have been identified in individuals with leukoencephalopathy [12, 13], but not yet in subjects with paragangliomas or pheochromocytomas. A *SDHAF2* loss-of-function mutation (p.Gly78Arg) has been reported in two unrelated families with head and neck paragangliomas [9, 14, 15]. In the tumors of affected individuals, this mutation was shown to impair flavinylation of SDHA. Additionally, in vitro experiments showed that the p.Gly78Arg mutant leads to complete loss of SDH activity, through impaired covalent flavinylation of SDHA and destabilization of the SDHAF2 protein. Subsequent studies in large cohorts of apparently sporadic paragangliomas and pheochromocytomas have failed to identify germline or somatic *SDHAF2* mutations, suggesting that mutations within *SDHAF2* may be rare [14, 15].

Recently, yeast studies showed that two LYR motif proteins Sdh6 (SDHAF1, human ortholog) and Sdh7 (SDHAF3, human ortholog) act in concert to promote the maturation of Sdh2 (SDHB, human ortholog) by shielding one or more of the three Fe-S clusters in Sdh2 from the deleterious effects of oxidants during assembly [10]. Mutations in the LYR motif of human *SDHAF1* were shown to attenuate interaction with iron-sulfur biogenesis components supporting a role for SDHAF1 in maturation of the holo-SDHB complex [16, 17]. L(I)YR motifs were also observed in SDHB itself in residues 44–46 (IYR) and 240–242 (LYR). The second motif is close to the binding site for SDHAF1 [17]. We therefore hypothesized that mutations within the newly identified SDH assembly factor, SDHAF3, may be associated with the pathogenesis of pheochromocytoma and/or paraganglioma syndromes. Furthermore, given SDHAF3 is involved in the maturation of SDHB, we hypothesized that mutations within either of these genes may impair this process.

## Methods

### Subjects and samples

DNA was extracted from peripheral blood leukocytes of 37 individuals (from 23 families) with germline *SDH* mutations (16 *SDHB,* 1 *SDHC* and 6 *SDHD* families) and 100 individuals with no known disease (ie. unaffected control population). Additionally, DNA was extracted from 15 fresh-frozen pheochromocytoma/paraganglioma samples of apparently sporadic origin, as well as 3 paraffin embedded pheochromocytoma/paraganglioma samples associated with familial disease. SDHB immunohistochemical assessment of available cases was performed, as previously described [18]. Informed consent was obtained for the collection and study of all samples, with approval for research being granted by the Northern Sydney Local Health District Human Research Ethics Committee (Kolling Neuroendocrine Tumour Bank Protocol #11011-361 M, Australian SHD Consortium Protocol #1103-101 M, and Kolling Institute Healthy Volunteers Bank Protocol HVBMC#14–06).

### Massively parallel sequencing

A custom gene panel (TruSeq® Custom Amplicon Assay, Illumina) was developed, encompassing our candidate gene - *SDHAF3* (NM_020186); as well as eight known pheochromocytoma/paraganglioma suseptibility genes (*MAX* [NM_002382], *SDHB* [NM_003000], *SDHC* [NM_003001], *SDHD* [NM_003002], *SDHAF2* [NM_017841], *RET* [NM_020975], *TMEM127* [NM_017849] and *VHL* [NM_000551]). The panel included the protein-coding exons and flanking intronic regions of each of the genes and was created using DesignStudio (Illumina). DNA libraries were prepared (using 250 ng of DNA from each sample) and sequenced on a MiSeq platform (using 2 × 150 bp paired end reads) according to the manufacturer’s instructions (Illumina). FASTQ files (containing reads and their base call quality scores) were generated for each sample; and alignment of reads (banded Smith-Waterman algorithm) and variant calling (GATK [19]) was processed by MiSeq Reporter (version 2.0, Illumina). Annotation of functional consequences to variant calls was performed using ANNOVAR (version 2013Jul [20]), which incorporates various in silico tools, including (but not limited to) PolyPhen-2, SIFT, MutationTaster. Visualisation of reads was performed using IGV (v2.1).

### Sanger sequencing

*SDHAF3* variants identified by massively parallel sequencing were confirmed by Sanger sequencing. Mutation analysis of the entire coding sequence, including exon-intron boundaries, was performed for the two exons of *SDHAF3*. Primer sequences were as follows: exon 1-forward (5′-gtctgccttccggttcacta-3′), exon 1-reverse (5′-gaacaggttgctgctctgttta-3′), exon 2-forward (5′-tccttaaccaaatgcttctgc-3′), exon 2-reverse (5′-tgatcttgatccatatactgcaa-3′). In some instances two rounds of PCR were required to amplify the DNA from paraffin embedded tissues, in such cases, each mutation was confirmed by sequencing of two independent PCRs.

### Studies in yeast

#### Strains and plasmids

*S. cerevisiae* strains used in this study were from a previous study [10]. Yeast cells were grown in synthetic complete or minimal media containing 2% raffinose and 0.2% glucose, unless indicated otherwise. To clone *SDHAF3* in yeast plasmids, the PCR-amplified *SDHAF3* ORF from a human cDNA library was ligated into pRS416 with *MET25* promoter and *CYC1* terminator. For *SDHAF3* and *SDH2* sequence variants, site-directed mutagenesis was carried out using Phusion high-fidelity DNA polymerase (Thermo Fisher Scientific). The full-length SDH2 with its own promoter and terminator were cloned into pRS416. Plasmids expressing Sdh2-His_6_Myc_2_ and Rip-Myc were from a previous study [21].

#### Co-immunoprecipitation

SDHAF3 immunoprecipitation with anti-SDHAF2 antibody was performed using protein A magnetic beads. Briefly, mitochondria were solubilized in 10 mM Tris-HCl (pH 7.4), 150 mM NaCl, 1 mM EDTA, 1% digitonin, and 1X protease inhibitor cocktail (Roche) for 30 min on ice. Supernatants after centrifugation at 14,000 x g were incubated with appropriate antibodies for 16 h at 4 °C. Protein A magnetic beads (New England Biolabs, Inc.) were added and incubated for 4 h at 4 °C. Beads were washed three times with 10 mM Tris-HCl (pH 7.4), 150 mM NaCl, 1 mM EDTA, 0.1% digitonin and 1 mM PMSF. Beads were resuspended in 2X SDS-PAGE sample buffer, which was subjected to immunoblotting.

#### Immunoblotting

Isolation of yeast mitochondria was performed using the method of Glick and Pon [22]. BN-PAGE was performed as described previously with mitochondrial lysates in 1% digitonin solution [23]. Samples were separated on 4–16% NativePAGE Bis-Tris gels (Life Technologies) and transferred to PVDF membrane for immunodetection. Anti-Sdh1, Sdh2 and Sdh3 were from a previous study [24]. Antibodies to Por1 were from Molecular Probes. Protein concentration was determined by the Bradford assay.

### In vitro studies in mammalian cells

#### Plasmid constructs and site-directed mutagenesis

Site-directed mutagenesis (QuikChange Lightning Site-Directed Mutagenesis Kit, Agilent) was used to produce the *SDHAF3* variant (p.Phe53Leu [c.157 T > C, NM_020186]) and *SDHB* mutants (p.Ala43Pro [c.127G > C, NM_003000], p.Arg46Gly [c.136C > G], p.Arg46Gln [c.137G > A], p.Cys101Tyr [c.302G > A], p.Ile127Ser [c.380 T > G], p.Pro197Arg [c.590C > G], p.Arg242His [c.725G > A]). The *SDHAF3* variant was generated in a commercially available plasmid, pCMV6-SDHAF3-Myc-DDK (RC204626, Origene); while the *SDHB* mutants were generated in a plasmid (pEGFP-N1; 6085–1, Clonetech) containing wild-type *SDHB*. Briefly, normal *SDHB* cDNA was generated from human adrenal total RNA and inserted into pEGFP-N1, using EcoRI and BamHI restriction sites, as previously described [25]. Sanger sequencing was used to confirm the presence of wild-type or variant sequences, and that they were in-frame with the respective tag.

#### Cell culture and transfections

Human embryonic kidney 293 (HEK293 [ATCC® CRL-1573™]) cells, cultured in DMEM with 10% fetal bovine serum, were seeded at 1.0 × 10^6^ cells/25 cm^2^ flask and left to settle overnight. For co-immunoprecipitation experiments, co-transfection of plasmids were performed 24 h post seeding using 7.5 μg of DNA (pCMV6-SDHAF3-Myc-DDK and pEGFP-N1-SDHB). For knockdown experiments, siRNA-mediated knockdown of SDHAF3 was performed by transfecting cells with a pre-designed siRNA (Ambion, Life Technologies, [4392420]) or negative control (scramble) siRNA (Ambion, Life Technologies, [AM4611]) at a final concentration of 12 nM for 48 h. All transfections were performed using Lipofectamine™ 2000 (Life Technologies) and Opti-MEM® (Life Technologies) according to the manufacturers’ instructions.

#### Co-immunoprecipitation

Twenty-four hours post transfection, cells were washed (PBS), pelleted and lysed using co-immunoprecipitation (Co-IP) buffer (20 mM Tris pH 7.5, 150 mM NaCl, 1 mM EGTA, 1 mM EDTA, 0.1% Triton X100), which was also used for whole cell lysates. Dynabeads® M-280 sheep anti-mouse IgG (Life Technologies) were incubated with either mouse IgG antibody (dilution 1:2000, Thermo-Fisher, Waltham, MA, USA) for negative control or mouse monoclonal anti-DDK (dilution 1:2000, OriGene [TA50011], MD, USA) for 2 h prior to washing; then incubated overnight with cell extracts at 4 °C under gentle rotation. Proteins not associated with DDK-tagged SDHAF3 were removed (3 × 10 min gentle agitation washes) using Co-IP lysis buffer with a higher salt concentration (500 mM NaCl). To remove immunoprecipitated material from beads, cell lysates were mixed with NuPAGE® LDS sample buffer (Invitrogen) and dithiothreitol and incubated at 95 °C for 5 min. Extracts were removed from beads, sonicated and separated by SDS-PAGE (4–12% NuPAGE Bis-Tris gels, Invitrogen) under reducing conditions. Proteins were transferred (nitrocellulose membrane) and the membrane blocked with 5% skim milk (in TBST) for 1 h at room temperature. The membranes were probed with the following antibodies: GFP (dilution 1:2000, Roche [11814460001], Basel, Switzerland), DDK (dilution 1:2000, OriGene [TA50011], MD, USA), GAPDH (dilution 1:5000, Cell Signaling [D16H11], MA, USA) and incubated overnight at 4 °C. Immunoblots were washed three times with TBST for 5–10 min and incubated with the relevant secondary antibody conjugated to horseradish peroxidase (HRP). Blots were then washed (three times in TBST for 5 min) and protein detected (ECL Plus Western Blotting Detection Reagent [GE Healthcare, Little Chalfont, UK]) on a LAS-3000 (Fujifilm, Brookvale, Australia).

#### Mitochondrial enzyme activity assays

Mitochondrial membrane fractions from HEK293 cells were extracted using QProteome Cell Compartment Kit according to the manufacturer’s instructions (Qiagen, Hilden, Germany) after two cold-PBS washes. Membrane lysates were homogenized by sonication and quantified using the PierceTM BCA protein assay (ThermoFisher [23225]).

Succinate dehydrogenase: Succinate dehydrogenase (SDH) and succinate:quinone oxidoreductase (SQR) activity assays were performed as previously described, with slight modifications [26]. The yeast enzyme was assayed using isolated mitochondria incubated in 40 mM potassium phosphate (pH 7.4) buffer with 0.5% Tween 80, 20 mM succinate and 20 μM antimycin A for 5 mins at room temperature. The reaction was initiated by adding 90 μM decylubiquinone for succinate-ubiquinol reductase activity or 120 μM phenazine methosulfate (PMS) for SDH with 120 μM 2, 6-dichlorophenolindophenol (DCPIP). The rate of reduction of DCPIP was measured spectrophotometrically at 600 nm for 5 mins. The human enzyme was assayed using 20 μg protein in phosphate buffer, pH 7.4, with 2 mM KCN, 1 μM antimycin A, 3 μM rotenone, pH 7.4 (in distilled water) with 20 mM) sodium succinate for succinate-DCPIP. Following 10 min incubation at room temperature, SDH was activated by 50 μM DCPIP and 1.6 mM of PMS. The decrease in absorbance at 600 nM over 5 mins due to reduction of DCPIP was measured as the conversion rate of succinate to fumarate, reflecting SDH activity (not involving the ubiquinol binding site).

Citrate synthase: Approximately 20 μg of protein was added to 470 μL of reaction mixture consisting (in final concentrations) 5 mM of KH_2_PO_4_, 45 mM of K_2_HPO_4_, 100 μM 5, 5′-dithio-bis-(2-nitrobenzoic acid) (DTNB) in distilled water at pH 7.4. Following 10 mins incubation for temperature equilibration at 37 °C, the reaction was activated by adding 5 μL of 10 mM acetyl CoA and 5 μL of oxaloacetic acid. Citrate synthase converts acetyl-CoA to citrate and CoA and the free thiol of CoA is quantified by reaction with DTNB, by monitoring the increase in absorbance at 412 nm over 10 mins.

### Statistical analyses

Exact statistics, specifically Pearson chi-square test, were used to compare allele counts in the disease-affected and disease-free cohorts, with *p* < 0.05 considered significant (SAS, v9.3). Yeast data were analyzed using Microsoft Excel 2011, with data presented as mean ± SD or mean ± SEM as indicated. Statistical significance was evaluated using Student’s test, with *p* < 0.05 considered significant.

## Results

### Massively parallel sequencing identifies germline variants arising in SDHB and SDHAF3 in the same individual

During validation of our targeted PC/PGL gene panel (MiSeq platform), we noted that one individual (S11_1) with a previously identified germline *SDHB* splice-site mutation (within intron 3 [IVS3]) also harbored a germline *SDHAF3* c.157 T > C (p.Phe53Leu) variant (rs62624461). Pheochromocytoma was first diagnosed in this individual at the age of 18 years, and subsequent recurrence and spinal metastasis was noted at 24 years of age. Although this *SDHAF3* variant has been identified in population studies (minor allele frequency [MAF] 0.0118 [1000 Genomes_Phase 3_ALL] and 0.0209 [Exome Aggregation Consortium, ExAC]), it is predicted to be damaging by several in silico tools (score of 0.777 [PolyPhen-2 v2.2.2r398], score of 0 [SIFT]). Since SDHAF3 was recently shown to be involved in mediating SDHB maturation [10], we determined the prevalence of the *SDHAF3* c.157 T > C variant among other subjects either with SDH-related PC/PGL or apparently sporadic PC/PGL, in comparison with normal controls.

### Analysis of SDHAF3 c.157 T > C variant in a normal population

The frequency of *SDHAF3* c.157 T > C in our Australian population was determined by direct sequencing of 100 healthy controls (48% males) with no known familial association to SDH-related disease. Of 200 alleles assessed, 6 were found to exhibit the minor allele (c.157C [NM_020186], p.Phe53Leu, rs62624461), resulting in a minor allele frequency (MAF) of 0.0300, which is consistent with the MAF reported in ExAC (0.0209; *p* = 0.452) (Table 1). However, the MAF observed in our Australian population differs from that reported in the 1000 Genomes (Phase 3_ALL) population (0.0118; *p* = 0.038). Of note, the MAF observed in ExAC (*n* = 60,422) differs from that reported in the 1000 Genomes (Phase 3_ALL, *n* = 2504) population (*p* < 0.0001).

**Table 1:** Summary of *SDHAF3* c.157 T > C (p.Phe53Leu) variant analysis in familial and suspected sporadic pheochromocytoma and/or paraganglioma

Cohort	SDHAF3 p.Phe53 germline allele count (T)	SDHAF3 p.Phe53Leu germline allele count (C)	MAF	*p* value^a^ (Australian)	*p* value^a^ (1000Genomes^b^)	*p* value^a^ (ExAC^c^)
Disease-affected						
All disease-affected (familial and sporadic; *n* = 38; alleles = 76)	71	5	0.0658	0.300	0.003	0.022
Apparently sporadic (*n* = 15; alleles = 30)	27	3	0.1000	0.098	0.006	0.025
Unrelated SDH-mutation carrier with disease (*n* = 23; alleles = 46)	44	2	0.0435	0.646	0.106	0.251
Unrelated SDHB-mutation carrier with disease (*n* = 16; alleles = 32)	30	2	0.0625	0.603	0.057	0.144
Disease-free						
Australian (*n* = 100; alleles = 200)	194	6	0.0300			
1000Genomes_Phase 3_ALL^b^ (*n* = 2504; alleles = 5008)	4949	59	0.0118	0.038		
ExAC^c^ (*n* = 60,422; alleles = 120,844)	118,315	2529	0.0209	0.452	<0.0001	

^a^Pearson chi-square test; ^b^The 1000 Genomes Project Consortium (2015) A global reference for human genetic variation *Nature* 526:68–74; ^c^Lek et al. (2016) Analysis of protein coding genetic variation in 60,706 humans. *Nature* 536:285–291

### Analysis of SDHAF3 c.157 T > C in individuals with pheochromocytoma and/or paraganglioma

When looking at all disease-affected individuals (familial and sporadic, *n* = 38; Table 1), there is an increased prevalence (6.6% [MAF 0.0658]) of the *SDHAF3* c.157 T > C variant when compared to the larger populations of disease-free individuals, specifically 1000 Genomes (Phase 3_ALL) and ExAC populations (1.2% [MAF 0.0118, *p* = 0.003] and 2.1% [MAF 0.0209, *p* = 0.022], respectively). However, no statistically significant difference was observed when comparing the prevalence of the *SDHAF3* c.157 T > C variant in disease-affected individuals (familial and sporadic) to a smaller disesase-free population (Australian, 3.0% [MAF 0.0300, *p* = 0.300]; Table 1).

### Analysis of SDHAF3 c.157 T > C in individuals with apparently sporadic pheochromocytoma and/or paraganglioma

To further assess the potential role of *SDHAF3* in pheochromoctyomas and/or paragangliomas, 15 tumors of apparently sporadic origin were assessed for the presence of the *SDHAF3* c.157 T > C (Additional file 1: Table S2). Three samples were found to be heterozygous for this variant, giving a MAF of 0.1000 (Table 1), which was significantly different from the 1000 Genomes (Phase 3_ALL) (*p* = 0.006) and ExAC (*p* = 0.025) populations (Table 1) but did not differ when compared to the Australian population (*p* = 0.098). SDHB immunohistochemical assessment was performed in those cases where formalin-fixed paraffin embedded tissue was available (*n* = 9; Table S2), only one of which harbors the *SDHAF3* c.157 T > C variant (#9). SDHB immunohistochemistry was positive in all cases, with no apparent difference in staining intensity or pattern observed between the *SDHAF3* c.157 T > C variant case and the wild-type cases (Additional file 1: Table S2).

### Analysis of SDHAF3 c.157 T > C in individuals with SDH-related familial pheochromocytoma and/or paraganglioma

In addition to individual S11_1, an additional 22 unrelated individuals with *SDH* germline mutations and evidence of disease (i.e. presence of pheochromocytoma and/or paraganglioma) were assessed for the presence of *SDHAF3* c.157 T > C (Additional file 2: Table S1). Of these, one additional individual was found to carry *SDHAF3* c.157 T > C (S55). This individual (S55) had multiple head and neck paragangliomas, the first (glomus jugulare tumor) being resected at the age of 48. A nodule at the left carotid bifurcation was noted at age 49 (MRI) and has subsequently been monitored, with no evidence of enlargement to date. Taken together, of 23 individuals with *SDH*-related familial pheochromocytoma and/or paraganglioma, two were heterozygous for *SDHAF3* c.157 T > C, giving a MAF of 0.0435 (Table 1) which was not significantly different from our Australian population of healthy controls (*p* = 0.646) nor from the 1000 Genomes (Phase 3_ALL) (*p* = 0.106) or ExAC (*p* = 0.251) populations (Table 1). When only *SDHB*-mutated individuals from our cohort are considered, then co-carriage of *SDHAF3* c.157 T > C (MAF 0.0625) does not differ from the Australian (*p* = 0.603), 1000 Genomes (Phase 3_ALL) (*p* = 0.057) or ExAC (*p* = 0.144) populations (Table 1).

### Extension of SDHAF3 c.157 T > C analysis in family S11

In addition to individual S11_1, an additional 14 *SDHB* mutation carrying members of this family (S11) were assessed (Additional file 3: Table S3). The *SDHAF3* c.157 T > C variant was identified in an additional seven *SDHB* mutation carrying family members. Five individuals in this family have (to date) presented with pheochromocytoma and/or paraganglioma (S11_1, S11_2, S11_3, S11_4 and S11_5). Of those with germline *SDHB* mutation and *SDHAF3* variant (*n* = 7), three (43%) have developed pheochromocytomas or paragangliomas (S11_1, S11_2 and S11_3); while two (25%) of those with germline *SDHB* mutation and wild-type *SDHAF3* (*n* = 8) have developed paragangliomas (*p* = 0.47, Chi-squared test). Of note, the tumors of individuals S11_1, S11_2 and S11_4 all exhibited loss of the normal *SDHB* allele and retention of the mutated allele (*SDHB* IVS3 splice-site mutation). Additionally, immunohistochemical assessment demonstrated loss of SDHB staining in all three tumors from individuals S11_1, S11_2 and S11_4.

### Functional characterization of SDHAF3 p.Phe53Leu in yeast

**Fig. 1:**
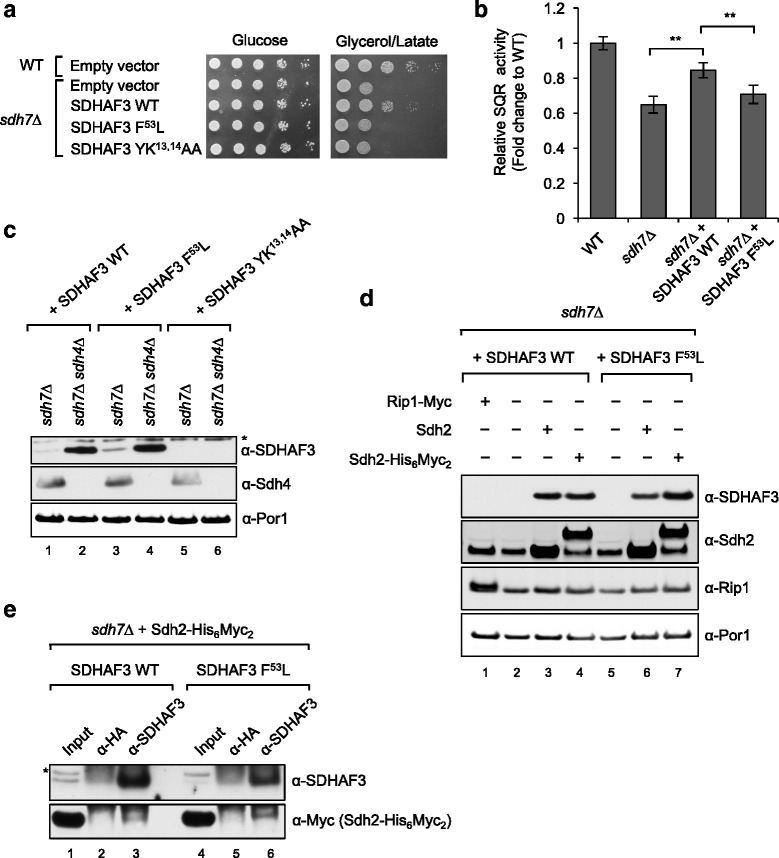
Phe53 substitution with Leu in SDHAF3 leads to impaired function of SDHAF3 in yeast. **a** Respiratory growth of yeast cells expressing a human Sdh7 ortholog, SDHAF3, and its sequences variants. Pre-cultured cells in synthetic complete (SC) media were serially diluted and then spotted on SC media containing different carbon sources, as indicated. Cells were incubated at 30 °C. **b** Relative succinate:quinone oxidoreductase (SQR) activity in isolated mitochondria. Mitochondria were isolated from cells grown until late-log phase in SC media plus 2% raffinose/0.2% glucose. Data are shown as mean ± SD (*n* = 3; **p < 0.05). **c** Steady-state levels of SDHAF3 proteins in *sdh4*∆ cells. Human SDHAF3 and its mutants under yeast *MET25* promoter were expressed from plasmids in cells. Por1 (porin) is a loading control. **d** Steady-state levels of SDHAF3 proteins in response to ectopic expression of Sdh2-His_6_Myc_2_. Rip1 is the Fe/S cluster subunit of *bc1* complex. **e** Co-immunoprecipitation of SDHAF3 was performed with mitochondrial isolated from cells expressing Sdh2-His_6_Myc_2_ using anti-SDHAF3 antibody and protein A magnetic beads. Anti-HA antibody is a negative control. *, non-specific reactivity; Input, 1% of total lysates

To test whether the substitution of p.Phe53 residue with Leu is pathogenic, we exploited *S. cerevisiae* as a model organism to conduct biochemical and molecular biological characterization of the p.Phe53Leu variant. The expression of wild-type (WT) SDHAF3 in yeast cells lacking *Sdh7*, a yeast ortholog of *SDHAF3*, enhanced respiratory growth of mutant cells; however, the SDHAF3 p.Phe53Leu variant failed to fully rescue the respiratory growth defect (Fig. 1a). Cells lacking Sdh7 exhibit a modest diminution in succinate:quinone oxidoreductase (SQR) activity [10]. Interestingly, the SDHAF3 p.Phe53Leu variant did not restore SQR activity in *sdh7*∆ cells, in contrast to the effect of WT SDHAF3 (Fig. 1b), suggesting that the p.Phe53Leu substitution in *SDHAF3* is a hypomorphic mutation that contributes to SDH deficiency.

Since SDHAF3 p.Phe53Leu appeared to be impaired functionally, we first examined whether expression of SDHAF3 p.Phe53Leu is compromised. We found that steady-state levels of SDHAF3 p.Phe53Leu were equal to those of WT SDHAF3 in *sdh7*∆ cells, consistent with stable expression of SDHAF3 p.Phe53Leu (Fig. 1c. Lane 1 and 3). Meanwhile, substitution of residues in the LYR motif (p.Tyr13Ala and p.Arg14Ala) dramatically decreased SDHAF3 levels (Fig. 1c. Lane 5). Since SDHAF3 p.Phe53Leu appeared to be well expressed compared to WT SDHAF3 in *sdh7*∆ cells, we next interrogated whether SDHAF3 p.Phe53Leu was competent to interact with Sdh2 (yeast ortholog of *SDHB*) during SDH assembly. Previously, we showed that the steady-state levels of Sdh7, increased in cells lacking the membrane anchor domain of SDH as a consequence of the enhanced interaction between Sdh7 and an Sdh1/Sdh2 pre-stalled intermediate (Sdh1, yeast ortholog of SDHA) [10]. Therefore, we measured SDHAF3 levels in *sdh4*∆ cells where an Sdh1/Sdh2 intermediate accumulates. SDHAF3 p.Phe53Leu as well as WT SDHAF3 accumulated in *sdh4*∆ cells (Fig. 1c. Lane 2 and 4), suggesting that SDHAF3 p.Phe53Leu may be capable of interacting with Sdh2. Recently, we observed that Sdh7 levels increased in response to an ectopic expression of Sdh2 in the presence of endogenous Sdh2 (data not shown). Therefore, we tested whether WT SDHAF3 and SDHAF3 p.Phe53Leu levels would also increase when Sdh2 is overexpressed in yeast cells. Indeed, an ectopic expression of Sdh2 or Sdh2-His_6_Myc_2_ in *sdh7*∆ cells harboring human SDHAF3 alleles resulted in increased levels SDHAF3, regardless of the p.Phe53Leu variation; however, the expression of Myc-tagged Rip1, a target of another LYR motif protein, Mzm1, failed to do so (Fig. 1d). We exploited this phenotype to further confirm the physical interaction between SDHAF3 p.Phe53Leu and Sdh2. We performed immunoprecipitation of SDHAF3 p.Phe53Leu with mitochondrial lysates from *sdh7*∆ cells wherein Sdh2-His_6_Myc_2_ is exogenously expressed. Indeed, Sdh2-His_6_Myc_2_ was co-precipitated with SDHAF3 p.Phe53Leu by anti-SDHAF3 antibodies, indicating that SDHAF3 p.Phe53Leu can interact with Sdh2 (Fig. 1e).

### Functional characterization of SDHAF3 p.Phe53Leu in mammalian cells

**Fig. 2:**
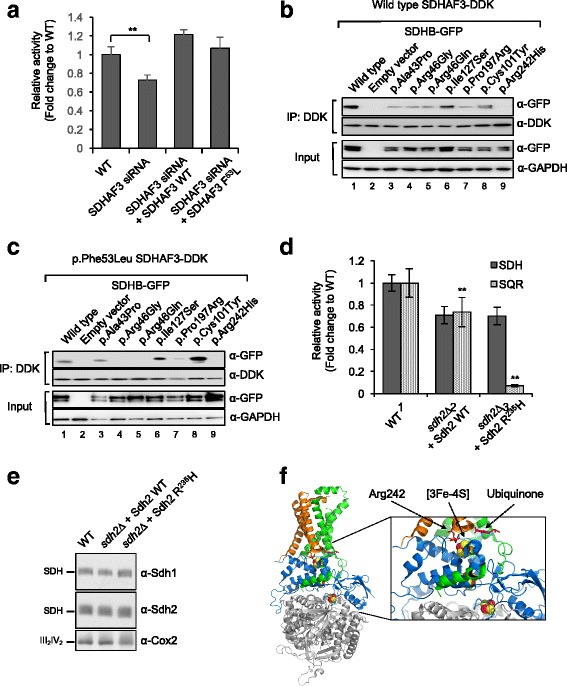
Phe53 substitution with Leu in SDHAF3 leads to impaired function of SDHAF3 in mammalian cells. **a** SDHAF3 was knocked down in HEK293 cells, using siRNA, to examine effects of SDHAF3 p.Phe53Leu on SDH (succinate hydrogenase) activity. Data shown as mean ± SD (*n* = 3; **p < 0.05). SDHAF3 interacts with SDHB in vitro*.* SDHAF3-SDHB complexes pulled down by DDK-tagged SDHAF3, following 24 h co-transfection with GFP-tagged SDHB in HEK293 cells, were immunoblotted for GFP and DDK. Stability of SDHB expression was assessed (whole cell lysates), following 24 h co-transfection with DDK-tagged SDHAF3 and GFP-tagged SDHB in HEK293 cells, when immunoblotted for GFP and GAPDH. **b** Interaction of wildtype SDHAF3 with wildtype SDHB was evident (Lane 1); complete abrogation of this interaction occurred with SDHB p.Arg242His (Lane 9); reduced interaction was observed for all other mutants (Lanes 3–5, 7–8) with exception of SDHB p.Ile127Ser whose interaction was unaffected. Stable SDHB expression was observed in whole cell lysates. **c** Introduction of SDHAF3 p.Phe53Leu with wildtype SDHB leads to reduced SDHAF3-SDHB interaction (Lane 1); complete loss of interaction was observed with SDHB p.Arg242His (Lane 9), p.Arg46Gly (Lane 4) and p.Arg46Gln (Lane 5); interaction with SDHB p.Ala43Pro and p.Pro197Arg was unaffected; while binding was enhanced with SDHB p.Ile127Ser and p.Cys101Tyr. Stable SDHB expression was observed in whole cell lysates. **d** Relative SDH and succinate:quinone oxidoreductase (SQR) activity with Sdh2 R^235^H in isolated mitochondria (from cells grown until late-log phase in SC media plus 2% raffinose/0.2% glucose). Data shown as mean ± SD (*n* = 3; **p < 0.05). **e** Blue native-PAGE analysis of mitochondrial lysates to visualize mature SDH complexes. III_2_IV_2_, a supercomplex consisting of complex III and complex IV, as loading control. **f** Porcine SDH (PDB: 1ZOY). SDHA (*gray*); B (*blue*); C (*green*); D (*Brown*); Fe-S centers (*red and yellow spheres*); Ubiquinone in the Q binding site (*magenta stick*); Arg242, corresponding to Arg235 in yeast (*red stick*)

To assess the effect of the SDHAF3 p.Phe53Leu variant in mammalian cells, SDHAF3 was knocked down in HEK293 cells using siRNA, and the effects on SDH (succinate dehydrogenase activity) measured. A reduction in SDH activity was observed in SDHAF3 knockdown cells compared to WT cells (*p* = 0.0132; Fig. 2a). Following SDHAF3 knockdown, re-introduction of WT SDHAF3 lead to restored SDH activity (Fig. 2a). Further to this, the p.Phe53Leu variant was also able to restore SDH activity to the same extent as the WT SDHAF3 (*p* = 0.1486; Fig. 2a). Western blotting of SDHB failed to show any significant differences in expression on re-introduction of the WT SDHAF3 and p.Phe53Leu variant, demonstrating that the observed effects on SDH activity were SDHAF3 dependent (data not shown).

Physical interaction between SDHB and SDHAF3 was validated in mammalian cells by co-immunoprecipitation of overexpressed SDHB (GFP-tagged) and SDHAF3 (DDK-tagged) in HEK293 cells. Similar to our observations in yeast, WT SDHB and WT SDHAF3 were shown to interact (Fig. 2b). This interaction was impaired with the introduction of the SDHAF3 p.Phe53Leu variant (Fig. 2c).

Further assessment of the interaction between SDHB and SDHAF3 was carried out using clinically relevant SDHB mutants. Introduction of SDHB mutants with WT SDHAF3 impaired normal SDHB-SDHAF3 interaction to varying degrees (Fig. 2b). Interestingly, complete abrogation of the SDHB-SDHAF3 interaction was seen with the SDHB p.Arg242His mutant; reduced interaction was observed for all other mutants, with the exception of the SDHB p.Ile127Ser mutant that appeared to be unaffected. Coupled with the stable SDHB expression observed in whole cell lysates (Fig. 2b), the complete loss of interaction observed on introduction of the SDHB p.Arg242His mutant is highly suggestive of p.Arg242 being a putative interaction site for SDHAF3.

The interaction observed between WT SDHB and SDHAF3 p.Phe53Leu was also impaired, to varying degrees, with the introduction of SDHB mutants (Fig. 2c). Complete loss of interaction was not only observed with the SDHB p.Arg242His mutant but also with mutants affecting residue 46 (p.Arg46Gly and p.Arg46Gln). Interestingly, enhanced binding was observed with SDHB p.Ile127Ser and p.Cys101Tyr mutants; whereas no effect on interaction was evident with SDHB p.Ala43Pro and p.Pro197Arg mutants. Stable expression of SDHB in whole cell lysates (Fig. 2c) indicates these observed changes in SDHB levels, following immunoprecipitation with SDHAF3, are reflective of changes to the SDHB-SDHAF3 interaction. Complete abrogation of SDHB-SDHAF3 interaction, following introduction of SDHB p.Arg46Gly and p.Arg46Gln mutants with SDHAF3 p.Phe53Leu (Fig. 2c), indicates that residue 46 of SDHB may also be involved in binding SDHAF3.

Additional studies in yeast revealed that Sdh2 p.Arg235His, which corresponds to the SDHB p.Arg242His mutation, resulted in impairment of SDH function. Compared to WT Sdh2, *sdh2*∆ cells expressing Sdh2 p.Arg235His exhibited reduced SQR activity (Fig. 2d). Interestingly, the substitution of p.Arg235 with His did not affect SDH activity (Fig. 2d) and SDH assembly (Fig. 2e). The 3Fe-4S cluster in SDHB is in close juxtaposition to p.Arg242 (Fig. 2f). The 3Fe-4S cluster in SDHB is essential for electron transfer to ubiquinone in the Q binding site in between SDHB and SDHC. Previously, we have shown that SDHAF3 confers protection on SDHB against reactive oxygen species (ROS) during SDHB maturation with Fe-S clusters [10]. Given that p.Arg242 in SDHB (p.Arg235 in Sdh2) is critical for the interaction between SDHB and SDHAF3 (Fig. 2b, lane 9), it is possible that the 3Fe-4S cluster may become more susceptible to ROS-related damage in SDHB p.Arg242His mutants. However, we cannot completely rule out the possibility that the Q binding site is altered in cells harboring SDHB p.Arg242His.

## Discussion

In this study, we have identified a variant in the SDH assembly factor 3 (*SDHAF3*, c.157 T > C [p.Phe53Leu]) that may be associated with increased prevalence of pheochromocytoma and/or paraganglioma (PC/PGL). Our studies in yeast have confirmed this to be a hypomorphic variant, leading to reduced SQR activity. Furthermore, our in vitro studies in human cells show that SDHAF3 interacts with SDHB (residues 46 and 242), and that interaction between SDHAF3 p.Phe53Leu and SDHB is impaired.

SDH plays an integral role in both the tricarboxylic acid cycle and electron transport chain. Germline mutations within any of its four subunits (SDHA, B, C and D) have been associated with development of a number of tumors, including pheochromocytoma and/or paraganglioma, gastrointestinal stromal tumors, renal cancer, and pituitary adenomas [1]. Recently, SDH assembly factors (SDHAF1–4) have been identified as playing a role in maturation of individual SDH subunits and assembly of the functioning SDH complex as a whole [27]. To date, loss-of-function mutations in *SDHAF1* (biallelic) and *SDHAF2* have been associated with infantile leukoencephalopathy [12, 13] and head and neck paragangliomas [9, 14, 15], respectively. More recently, the yeast orthologs for SDHAF1 (Sdh6) and SDHAF3 (Sdh7) were shown to shield the Fe-S clusters of the yeast ortholog for SDHB (Sdh2), thereby promoting maturation of SDHB [10]. In human cells, SDHAF1 was shown to associate with iron-sulfur cluster biogenesis components suggesting a role for SDHAF1 in mediating Fe-S cluster insertion [16, 17]. Taken together, we hypothesized that mutations within the newly identified SDH assembly factor, *SDHAF3*, may be associated with the pathogenesis of pheochromocytoma and/or paraganglioma syndromes.

In this study, we identified a *SDHAF3* c.157 T > C (p.Phe53Leu) variant in familial and sporadic cases of PC/PGL, observing a minor allele frequency (MAF) of 0.0658. Although this variant (rs62624461) is reported with a MAF of 0.0209 in the Exome Aggregation Consortium (ExAC) database (http://exac.broadinstitute.org/), reflecting exome variant data from 60,422 individuals, the prevalence is significantly higher in PC/PGL (6.6% versus 2.1%, *p* = 0.022). This prompted us to perform additional studies, to clarify the role that the SDHAF3 p.Phe53Leu variant may play in the pathogenesis of PC/PGL. Through yeast studies we were able to show that introduction of the SDHAF3 p.Phe53Leu variant, into *Sdh7* null yeast (ortholog of *SDHAF3* in humans) resulted in impaired function, observed by its failure to fully restore SDH activity when expressed in *Sdh7* null yeast relative to wild-type (WT) SDHAF3. Taken together, these findings indicate that although SDHAF3 p.Phe53Leu is at best a very low penetrance allele for PC/PGL per se, it may play a modifying role as observed by its hypomorphic activity. Hypomorphic alleles of SDHAF3 may contribute to the pathology in SDH-deficient tumors with residual SDH subunits, or alternatively through a secondary unidentified function of SDHAF3. In this study, two PC/PGL tumors from patients harboring germline *SDHB* (IVS3 splice-site) mutation and *SDHAF3* (c.157 T > C) variant showed loss of SDHB staining by immunohistochemistry. This raises the question of how *SDHAF3* c.157 T > C can play a role in PC/PGL tumorigenesis, in SDH-deficient tumors. Clearly, by the time that inactivation of both *SDHB* alleles has occurred in the tumor, *SDHAF3* c.157 T > C presumably has no additional role, as SDHAF3 appears to interact specifically with SDHB. Nevertheless, we conjecture that the germline presence of this hypomorphic *SDHAF3* c.157 T > C allele may over time lead to instability of SDH. Further, as *SDHB* is a known tumor suppressor and hence requires inactivation of both alleles for tumorigenesis, the timeframe between *SDHB* germline (first hit) and somatic loss of the normal *SDHB* allele (second hit) provides a means by which the *SDHAF3* c.157 T > C allele could act. Further research is needed to resolve this issue.

To further understand the role of SDHAF3, and the impact of p.Phe53Leu in greater detail, we assessed its ability to interact with SDHB. Our in vitro studies in human cells, confirmed previous findings in yeast, [10] with wild-type SDHAF3 shown to interact with SDHB. Interestingly, this interaction was attenuated on introduction of the p.Phe53Leu variant. We wanted to assess SDHAF3-SDHB interaction further by introducing clinically relevant SDHB mutations. Interaction between wild-type SDHAF3 and SDHB p.Arg242His mutant was not observed, implicating this region of SDHB as a direct binding site for SDHAF3. Maio et al. (2015) recently demonstrated that SDHAF1 interacts with SDHB with contacts between SDHB residues 146–153, 183–185 and 198–202 [17]. Thus, two LYR-motif proteins, SDHAF1 and SDHAF3, may associate with different contact sites on SDHB during maturation of SDHB with Fe-S clusters.

This finding is supported by observations in yeast, whereby Sdh6 and Sdh7 (orthologs of human SDHAF1 and SDHAF3, respectively) have been shown to interact with Sdh2 (ortholog of human SDHB) [10], although the specific binding site(s) in yeast Sdh2 were not identified. Our study shows that SDHAF3, in fact, is a direct binding partner for the LYR motif of SDHB (p.240–242). Reduced interaction was observed in all other SDHB mutants, with the exception of p.Ile127Ser in which interaction between SDHAF3 and SDHB appeared to be unaffected, indicating that this residue has no bearing on SDHB interaction with SDHAF3.

On introduction of the SDHAF3 p.Phe53Leu variant, SDHAF3-SDHB interaction was completely lost for SDHB p.Arg46Gln and p.Arg46Gly mutants, implicating residue 46 (contained within an IYR binding site [p.44–46]) as another region of SDHB that may interact with SDHAF3. Although these two regions are spatially separated in two distinct sub-domains, SDHAF3 may in fact bind the L(I)YR motifs residues in each of these domains. Alternatively, the impaired binding may arise from secondary consequences of the p.Arg46 mutation. Interestingly, our previous structural modeling of these *SDHB* mutations had not identified the functional impact on SDHB, as both glycine and glutamine are capable of fitting within the space left by arginine, and the electron path is not nearby [25]. The findings of our current study suggest that mutations affecting residue 46 of SDHB are pathogenic via preventing maturation of SDHB. A similar effect was noted by Maio et al. (2014), whereby the SDHB p.Arg46Gln mutation did not impair SDHB interaction with HSC20, although reduced binding to the HSC20 complex and SDHA were noted, suggestive of an effect on formation of a mature SDH complex [16].

Interestingly, introduction of the SDHAF3 p.Phe53Leu variant resulted in a stronger SDHAF3-SDHB interaction in the presence of the SDHB p.Cys101Tyr mutant. Since Cys101 is a ligand to the 2Fe-2S center in the N-terminal domain of SDHB, the enhanced interaction is suggestive that SDHAF3 interacts with apo-SDHB. Consistent with this prediction is the observation that overexpression of *SDH2* in yeast lacking Sdh1 results in a profound stabilization of Sdh7 (SDHAF3) (U.N., unpublished data). SDHAF3 may, therefore, contribute to the Fe-S insertion process in SDHB.

## Conclusion

Our studies have revealed novel insights into the biogenesis of SDH, uncovering a vital interaction between SDHAF3 and SDHB. We have shown that SDHAF3 interacts directly with SDHB (residue 242 being key to this interaction), and that a variant in *SDHAF3* (c.157 T > C [p.Phe53Leu]) appears to be more prevalent in individuals with pheochromocytomas and/or paragangliomas, and is hypomorphic via impaired interaction with SDHB. Further studies of larger numbers of PC/PGL will, however, be required to fully clarify the role of *SDHAF3* (c.157 T > C [p.Phe53Leu]) in the pathogenesis of PC/PGL.

## Additional files

Additional file 1: Table S2. Summary of SDHAF3 c.157 T > C (p.Phe53Leu) variant analysis in pheochromocytoma and/or paraganglioma of suspected sporadic origin. Fifteen pheochromoctyomas and/or paragangliomas of apparently sporadic origin were assessed (using massively parallel sequencing and/or Sanger sequencing) for the presence of *SDHAF3* c.157 T > C. (PDF 119 kb)

Additional file 2: Table S1. Summary of SDHAF3 c.157 T > C (p.Phe53Leu) variant analysis in familial SDH-associated individuals. Twenty-three unrelated individuals with *SDH* germline mutations and evidence of disease (i.e. presence of pheochromocytoma and/or paraganglioma) were assessed (using massively parallel sequencing and/or Sanger sequencing) for the presence of *SDHAF3* c.157 T > C. (PDF 236 kb)

Additional file 3: Table S3. Summary of SDHAF3 c.157 T > C (p.Phe53Leu) variant analysis in Family S11. In addition to individual S11_1, an additional 14 *SDHB* mutation carrying members of this family (S11) were assessed (using massively parallel sequencing and/or Sanger sequencing) for the presence of *SDHAF3* c.157 T > C. (PDF 230 kb)

## Abbreviations

Co-IP: co-immunoprecipitation
ExAC: Exome Aggregation Consortium
HEK293: human embryonic kidney 293 cells
HIF: hypoxia inducible factor
IGV: integrative genomics viewer
MAF: minor allele frequency
PC: pheochromocytoma
PGL: paraganglioma
SDH: succinate dehydrogenase
SDHAF3: succinate dehydrogenase assembly factor 3
SDHB: succinate dehydrogenase subunit B
SQR: succinate:quinone oxidoreductase
TCA: tricarboxylic acid
WT: wild type
