# Supplementary material for: Analysis of SDHAF3 in familial and sporadic pheochromocytoma and paraganglioma
Source: BMC Cancer. 2017 Jul 24;17:497. doi: 10.1186/s12885-017-3486-z (PMC5525311; doi:10.1186/s12885-017-3486-z)
Supplement: Supplementary file 1 — Summary of SDHAF3 c.157 T > C (p.Phe53Leu) variant analysis in pheochromocytoma and/or paraganglioma of suspected sporadic origin. Fifteen pheochromoctyomas and/or paragangliomas of apparently sporadic origin were assessed (using massively parallel sequencing and/or Sanger sequencing) for the presence of SDHAF3 c.157 T > C. (PDF 119 kb) [file 12885_2017_3486_MOESM1_ESM.pdf]

Table S2. Summary of *SDHAF3* c.157T>C (p.Phe53Leu) variant analysis in pheochromocytoma and/or paraganglioma of suspected sporadic origin

Tumour ID	Tumor Type	Primary Germline Mutation	Somatic *SDHAF3* c.157T>C Status	Germline *SDHAF3* c.157T>C Status	SDHB IHC
1	PC (benign, 23 years)	none known[a]	WT	nd	positive[b]
2	PC (benign, 45years)	none known[a]	c.157T>C (heterozygous)	c.157T>C (heterozygous)	na
3	PC (benign, 47 years)	none known[a]	WT	nd	positive[b]
4	PC (benign, 67 years)	none known[a]	WT	nd	na
5	PC (benign, 58 years)	none known[a]	c.157T>C (heterozygous)	c.157T>C (heterozygous)	na
6	PC (benign, 75 years)	none known[a]	WT	nd	na
7	PC (benign, 68 years)	none known[a]	WT	nd	positive[b]
8	PC (benign, 64 years)	none known[a]	WT	nd	positive[b]
9	PC (benign, 47 years)	none known[a]	c.157T>C (heterozygous)	c.157T>C (heterozygous)	positive[b]
10	PC (benign, 67 years)	none known[a]	WT	nd	positive[b]
11	PGL (benign, 36 years)	none known[a]	WT	nd	positive[b]
12	PC (malignant, 56 years)	none known[a]	WT	nd	positive[b]
13	PC (malignant, 52 years)	none known[a]	WT	nd	positive[b]
14	PC (malignant, 49 years)	none known[a]	WT	nd	na
15	PC (malignant, 37 years)	none known[a]	WT	nd	na

Abbreviations: IHC - immunohistochemistry; na - not available; nd - not done; PC - pheochromocytoma; PGL - paraganglioma (extra adrenal thoracic/abdominal); WT - wild-type

[a] Germline tests included *SDHB* (all exons), *SDHD* (all exons), *RET* (exon 11 only), and *VHL* (all exons), and were performed using PCR and denaturing high performance liquid chromatography (dHPLC) as previously described (Meyer-Rochow et al., 2010).

[b] SDHB IHC performed on whole section at the time of diagnosis and/or tissue microarray.
