# Supplementary material for: Analysis of SDHAF3 in familial and sporadic pheochromocytoma and paraganglioma
Source: BMC Cancer. 2017 Jul 24;17:497. doi: 10.1186/s12885-017-3486-z (PMC5525311; doi:10.1186/s12885-017-3486-z)
Supplement: Supplementary file 2 — Summary of SDHAF3 c.157 T > C (p.Phe53Leu) variant analysis in familial SDH-associated individuals. Twenty-three unrelated individuals with SDH germline mutations and evidence of disease (i.e. presence of pheochromocytoma and/or paraganglioma) were assessed (using massively parallel sequencing and/or Sanger sequencing) for the presence of SDHAF3 c.157 T > C. (PDF 236 kb) [file 12885_2017_3486_MOESM2_ESM.pdf]

Table S1. Summary of *SDHAF3* c.157T>C (p.Phe53Leu) variant analysis in familial SDH-associated individuals

Family ID	Individual ID	Tumor Details	Primary Germline Mutation	Somatic *SDHB* Mutation/Allele Status	Germline *SDHAF3* c.157T>C Status	Somatic *SDHAF3* c.157T>C/Allele Status
**S11**	1	PC (metastatic)	SDHB (splice-site)	IVS3 + loss normal allele	c.157T>C	c.157T>C + retention normal allele
**S50**	1	PGL	SDHB (nonsense)	nd	WT	nd
**S15**	1	PC (metastatic)	SDHB (splice-site)	nd	WT	nd
**S18**	1	PGL	SDHB (frameshift)	nd	WT	nd
**S24**	1	PGL	SDHB (splice-site)	nd	WT	nd
**S26**	1	PGL	SDHB (missense)	nd	WT	nd
**S27**	1	PGL	SDHB (frameshift)	nd	WT	nd
**S52**	1	HN PGL	SDHB (nonsense)	nd	WT	nd
**S55**	1	HN PGL	SDHB (frameshift)	nd	c.157T>C	nd
**S59**	1	HN PGL	SDHB (nonsense)	nd	WT	nd
**S25**	1	HN PGL	SDHB (missense)	nd	WT	nd
**S10**	1	PGL (metastatic)	SDHB (missense + splice-site)	nd	WT	nd
**S16**	1	PGL (metastatic)	SDHB (splice-site)	nd	WT	nd
**S20**	1	PC	SDHB (splice-site)	nd	WT	nd
**S37**	1	PC (metastatic)	SDHB (splice-site)	nd	WT	nd
**S44**	1	PC (metastatic)	SDHB (frameshift)	nd	WT	nd
**S69**	1	PGL	SDHC (nonsense)	nd	WT	nd
**S82**	1	HN PGL	SDHD (missense)	nd	WT	nd
**S83**	1	PGL	SDHD (missense)	nd	WT	nd
**S84**	1	HN PGL	SDHD (frameshift)	nd	WT	nd
**S88**	1	HN PGL	SDHD (frameshift)	nd	WT	nd
**S93**	1	PC + HN PGL	SDHD (nonsense)	nd	WT	nd
**S105**	1	HN PGL	SDHA (missense)	nd	WT	nd

Abbreviations: IVS - intervening sequence; nd - not done; PC - pheochromocytoma; PGL - paraganglioma (extra adrenal thoracic/abdominal); HNPGL (head and neck paraganglioma); WT - wild-type
