# Supplementary material for: Analysis of SDHAF3 in familial and sporadic pheochromocytoma and paraganglioma
Source: BMC Cancer. 2017 Jul 24;17:497. doi: 10.1186/s12885-017-3486-z (PMC5525311; doi:10.1186/s12885-017-3486-z)
Supplement: Supplementary file 3 — Summary of SDHAF3 c.157 T > C (p.Phe53Leu) variant analysis in Family S11. In addition to individual S11_1, an additional 14 SDHB mutation carrying members of this family (S11) were assessed (using massively parallel sequencing and/or Sanger sequencing) for the presence of SDHAF3 c.157 T > C. (PDF 230 kb) [file 12885_2017_3486_MOESM3_ESM.pdf]

Table S3. Summary of *SDHAF3* c.157T>C (p.Phe53Leu) variant analysis in Family S11

Family ID	Individual ID	Tumor Details	Primary Germline Mutation	Somatic *SDHB* Mutation/Allele Status	Germline *SDHAF3* c.157T>C Status	Somatic *SDHAF3* c.157T>C/Allele Status
**S11**	1	PC (18 years) (metastatic, 24 years)	SDHB (IVS3)	IVS3 + loss normal allele	c.157T>C (heterozygous)	c.157T>C + retention normal allele
	2	PC (metastatic, 59 years)	SDHB (IVS3)	IVS3 + loss normal allele	c.157T>C (heterozygous)	na
	3	PGL (30 years)	SDHB (IVS3)	nd	c.157T>C (heterozygous)	nd
	4	PGL (11 years)	SDHB (IVS3)	IVS3 + loss normal allele	WT	WT
	5	PGL (34 years)	SDHB (IVS3)	nd	WT	nd
	6	No evidence of PC/PGL (66 years)	SDHB (IVS3)	nd	c.157T>C (heterozygous)	nd
	7	No evidence of PC/PGL (22 years)	SDHB (IVS3)	nd	c.157T>C (heterozygous)	nd
	8	No evidence of PC/PGL (62 years)	SDHB (IVS3)	nd	c.157T>C (heterozygous)	nd
	9	No evidence of PC/PGL (57 years)	SDHB (IVS3)	nd	c.157T>C (heterozygous)	nd
	10	No evidence of PC/PGL (35 years)	SDHB (IVS3)	nd	WT	nd
	11	No evidence of PC/PGL (34 years)	SDHB (IVS3)	nd	WT	nd
	12	No evidence of PC/PGL (29 years)	SDHB (IVS3)	nd	WT	nd
	13	No evidence of PC/PGL (26 years)	SDHB (IVS3)	nd	WT	nd
	14	No evidence of PC/PGL (23 years)	SDHB (IVS3)	nd	WT	nd
	15	No evidence of PC/PGL (66 years)	SDHB (IVS3)	nd	WT	nd

Abbreviations: IVS - intervening sequence; na - not able to amplify; nd - not done; PC - pheochromocytoma; PGL - paraganglioma (extra adrenal thoracic/abdominal); WT - wild-type
